# Lived experiences of patients using positive airway pressure (PAP) therapy: a nested phenomenological study within the 3DPiPPIn randomised controlled trial

**DOI:** 10.1136/bmjopen-2024-093622

**Published:** 2025-05-06

**Authors:** Stephanie K Mansell, Francesca Gowing, Stephen T. Hilton, Eleanor Main, Swapna Mandal, Silvia Schievano, Cherry Kilbride

**Affiliations:** 1 University College London Institute of Cardiovascular Science, London, UK; 2 Therapy Services, Royal Free London NHS Foundation Trust, London, UK; 3 UCL School of Pharmacy, London, UK; 4 Physiotherapy, University College London Institute of Child Health, London, UK; 5 Thoracic Medicine, Royal Free London NHS Foundation Trust, London, UK; 6 UCL Medical School, London, UK; 7 Institute of Cardiovascular Science, University College London Institute of Cardiovascular Science, London, UK; 8 Brunel University Division of Physiotherapy, London, UK

**Keywords:** SLEEP MEDICINE, QUALITATIVE RESEARCH, Protocols & guidelines

## Abstract

**Background:**

Sleep disordered breathing is a chronic condition often requiring patient commitment to positive airway pressure (PAP) therapy. Understanding the lived experience of PAP therapy users is crucial for clinicians to support successful treatment and identify research priorities. There is a lack of evidence in this area, and published data predominantly explore the negative experiences of PAP. This study aims to explore the lived experiences of patients using PAP therapy.

**Methods and analysis:**

This study employs a phenomenological approach, appropriate for researching human experiences where there is little existing research. Heideggerian theory underpins the research, recognising that the researcher’s beliefs influence meaning, allowing for rich analysis of the lived experience.

Participants will be recruited from a randomised controlled trial investigating the medium-term clinical impact of customised interfaces for patients requiring PAP therapy. Purposive sampling will be used to seek representation from various demographics, with a maximum of 30 participants.

Data collection will be via 1:1 semistructured interviews. Data will be analysed using Braun and Clarke’s six-phase reflexive thematic analysis. Data will be analysed inductively through an interpretivism lens. Data will be managed with computer-assisted qualitative data analysis software.

**Ethics and dissemination:**

This protocol has been approved by the Hampshire B Research Ethics Committee (REC reference: 22/SC/0405). Results will be disseminated to healthcare professionals and patients through conferences, open-access journals, newsletters, the study webpage, infographics, animations, social media and healthcare awards. Tracy’s eight ‘big tent’ criteria for excellent qualitative research are comprehensive and encompassing, and this protocol has aimed to meet the criteria. The Consolidated Criteria for Reporting Qualitative Research has also been used. The findings of this study will contribute to a more holistic understanding of the lived experience of PAP therapy users, informing clinical practice and future research.

**Trial registration number:**

ISRCTN74082423.

**STRENGTHS AND LIMITATIONS OF THIS STUDY**

- Uses a phenomenological approach, which is well-suited for exploring the lived experiences of individuals, providing deep insights into the human aspects of positive airway pressure therapy.
- The use of Heideggerian theory allows for a rich and nuanced analysis by acknowledging the influence of the researcher’s beliefs on the interpretation of data.
- Uses Braun and Clarke’s six-phase reflexive thematic analysis, a robust and systematic method for analysing qualitative data, ensuring thorough and reflective data interpretation.
- The quality of data is dependent on the participants’ willingness and ability to articulate their experiences, which can vary significantly and could affect the richness of the data.
- The influence of the researcher’s beliefs, while acknowledged, may still introduce subjective bias into the interpretation of the qualitative data.

## Introduction

 Sleep disordered breathing is a long-term condition often treated with positive airway pressure (PAP) therapy, which requires ongoing commitment from patients to benefit from treatment. Comprehending the lived experience of patients undergoing PAP therapy could help clinicians support patients in achieving treatment success. Additionally, understanding the lived experience could help identify future research priorities. Furthermore, engaging patients in such technologies is critical as technologies improve.

A comprehension of the lived experience of patients undergoing PAP therapy could facilitate clinicians to support patients in successful treatment use. The addition of PAP therapy is disruptive to not just the individuals’ lives but also those around them.^1^ Untreated obstructive sleep apnoea (OSA) impacts wider society,^2^ and this wider impact goes unrecognised in the design of patient pathways. Furthermore, as patient pathways evolve, aiming to become more efficient, engaging patients in and appreciating their acceptance of such pathways is critical. A systematic interpretive literature review^1^ reported a paucity of information about the experiences of continuous PAP (CPAP) users from their perspective. Of the 22 studies included, only seven were qualitative in nature. The studies included had global representation, although all bar one were conducted in the Western world. The included studies represented patients with moderate to severe OSA. The duration of the included studies ranged from one night to 6 months. Six of the included studies represented patients who had discontinued treatment. There was a narrow focus of the research on factors that facilitated or hindered CPAP use. Thematic analysis of the included studies identified three themes related to experiences using CPAP: ‘*users’ beliefs about CPAP influence users’ experiences of CPAP’, ‘experiencing CPAP is investigated and reported as synonymous with experiencing difficulty’,* and ‘*spouse and family influence users’ experience of CPAP’*. Ward *et al*^1^ observed evidence of bias in reporting, with the research instruments used limiting exploration of negative experiences of CPAP. Brown *et al*^3^ focused on establishing reasons for non-concordance with PAP therapy in their systematic review and thematic synthesis. They report four themes: ‘*journey to PAP’, ‘discomfort from and around PAP’, ‘adapting to and using PAP’* and ‘*benefits from PAP’*. Brown *et al*^3^ acknowledge there is little existing qualitative research investigating the lived experience of PAP therapy. They reflected on the applicability of a biopsychosocial understanding of PAP therapy and suggested ways in which services could address barriers to PAP therapy concordance. More recently, in their content analysis, Simon *et al*^4^ highlighted the importance of understanding the lived experience in order to develop adherence-promoting interventions. The research to date has focused on PAP therapy adherence, and by not accounting for the whole lived experience, our understanding becomes constrained. This limited understanding could mean that current clinical pathways are misaligned with the lived experience. Qualitative research, particularly phenomenology, facilitates the amplification of the patient voice. There is a need for further qualitative research that aims to fully encompass the entirety of the lived experience of PAP therapy users.

### Aims

To explore the lived experiences of patients using PAP therapy.

## Methods and analysis

### Study design and theoretical framework

This qualitative study is nested within a randomised controlled trial (RCT). A phenomenological approach has been chosen for this qualitative research study as it is appropriate for researching human experiences where there is little research into the phenomenon of interest and to allow an increased insight into lived experiences.

The research will use a phenomenological approach underpinned by a Heideggerian theory.^5^ Phenomenology with a Heideggerian perspective recognises that the researcher’s beliefs influence meaning. Given that the primary researcher (SKM) is an experienced clinician in sleep and ventilation medicine, it will be challenging to bracket beliefs; hence, a phenomenological approach with a Heideggerian perspective is most appropriate. A Heideggerian phenomenological approach allows for rich analysis and facilitates the understanding of multiple facets of the lived experience, providing a more comprehensive ontological (the experience of existence experienced by individuals) perspective of Being.^5^ As such, Heideggerian phenomenology recognises the importance of everyday context, thus enriching the analysis and providing a holistic view of the lived experience (Being), such as daily lives, relationships and societal and environmental aspects of living. Existential exploration is a core component of Heideggerian phenomenology, and this existential lens facilitates an analysis that goes beyond description and delves into the fundamentals of Being. Key aspects prioritised within Heideggerian phenomenology are depth, context and participant perspectives. These components empower the researcher to uncover the essence of the lived experience.

### Sampling/recruitment

#### Participants

Participants will be recruited from those participating in both arms of an RCT (3DPiPPIn). The RCT has been designed as a pragmatic trial and is embedded into existing clinical pathways. The 3DPiPPIn trial investigates the medium-term clinical impact of customised interfaces for patients requiring PAP therapy. Patients recruited will be under a specialist sleep and ventilation team in a secondary/tertiary care setting.

#### Sample

The study will recruit participants from the intervention and control arms of the 3DPiPPIn RCT to gain a range of experiences. The study aims to recruit a maximum of 30 participants.^6^ Purposive sampling will be used to seek broad representation from a variety of demographics (age, sex, ethnicity and concordance with PAP therapy), aiming for the sample to represent maximum variation by key selected characteristics for this study.^7^

#### Inclusion criteria

- Patients participating in the 3DPiPPIn trial.
- Diagnosis of sleep-disordered breathing: Apnoea Hypopnoea Index (AHI)≥15.
- Patients naive to domiciliary PAP therapy.
- Age≥18 years.

#### Exclusion criteria

- Patients unable to provide informed consent.
- AHI<15.
- Excessive facial hair, which they are unwilling to shave.
- Age <18 years.
- Existing facial pressure ulcers.
- Unable to provide informed consent.
- Known allergy to silicone.
- Keloid scarring.
- Previous domiciliary PAP therapy.

### Data Handling and Analysis

#### Data collection and development of the topic guide

Data collection will be via 1:1 semistructured interviews. Participants will be given a choice between face to face interviews at the Royal Free Hospital or online via a virtual videoconferencing platform. Through piloting and feedback from patient and public involvement and engagement (PPIE) groups, it is recognised that a choice between Zoom and Microsoft Teams increases acceptability and access to participation. Thus, both platforms will be offered to participants. Face to face interviews will be recorded using Samson Go Mic (Samson Technologies Corp, New York, USA). Interviews conducted via virtual videoconferencing platforms will be audiovisually recorded. Semistructured 1:1 interviews have been chosen to allow for depth and breadth of response while allowing flexibility for participants. In keeping with the Heideggerian phenomenological approach, the interviews will be indepth and dialogical in nature. The researcher aims to ensure the depth of participants’ responses. Thus, when they provide descriptions, the researcher will prompt exploration through probing and encourage the use of examples to illuminate the descriptions.^8^ It is recognised in Heideggerian phenomenology that the subjective judgement of the researcher is valuable. However, the researcher will aim to ensure the participant’s perspective is the focus of the research and avoid leading questions. Reflection and clarification will ensure that participants’ meaning is understood. Active listening techniques will be deployed throughout the interviews to convey interest and enhance rapport. Interviews will be transcribed verbatim using a general data protection regulation (GDPR)-compliant transcription service (Typeout, UK), with transcripts reviewed by the participants for accuracy.

#### Topic guide

An initial topic guide was developed based on the research aim, clinical experience and literature from the systematic review, which was reviewed by the trial PPIE group. The topic guide is designed to ensure participants feel comfortable, build rapport and assure depth of response regarding the lived experience. Opening questions are broad, questions are grouped around particular topics and more sensitive questions are asked later in the interview once rapport has been established. The topic guide and interview process were piloted with one member of the PAG who was a current PAP therapy user. The pilot interview was transcribed verbatim using a transcription service (Typeout, UK). The pilot interview recording and transcription were reviewed by an experienced qualitative researcher (CK) who provided feedback and guidance on how the interview was conducted to ensure it was in keeping with the philosophical framework described above. The participant reviewed the transcripts for accuracy. Amendments to the topic guide were made accordingly, with the order of some questions adjusted. The topic guide is designed as an aide-mémoire for the researcher to ensure the depth and breadth required of a phenomenological approach. The topic guide is not intended to be limiting and aims to enhance rapport. Additional questions will be asked where needed to ensure that the data collected represents the participant’s lived experience. The topic guide includes prompts regarding expectations, health, societal impacts, work, leisure, lifestyle and sources of advice.

#### Data storage and confidentiality

Data storage aligns with the UK Data Protection Act 2018, which sets out the GDPR. University College London (UCL) accounts for both Zoom and Microsoft Teams will be used for the videoconferencing interviews. This ensures that data are both collected and stored securely. Participants will provide informed consent via an electronic consent form. Transcripts will be anonymised after participants have reviewed them for accuracy. Interview recordings will be kept until the transcription is complete and will subsequently be destroyed in line with the UK Data Protection Act 2018.

#### Data analysis

Multiple analytical approaches are possible when undertaking a phenomenological-based study. Braun and Clarke’s reflexive thematic analysis^9^ is a flexible and helpful method for analysis and can be used within several theoretical frameworks, including phenomenology.^10^ Reflexive thematic analysis is often considered to be more accessible than other analysis methods. Reflexive thematic analysis provides a rich and detailed account of data, allowing for deep exploration of the lived experience and is thus compatible with a Heideggerian phenomenological approach. Braun and Clarke’s reflexive thematic analysis supports both an inductive and deductive approach to data analysis. Inductive analysis is strongly linked with the data and allows the researcher to immerse themselves in it. Data will be analysed inductively with an interpretivism lens, which assumes the world is interpreted through our experiences and accounts for the impact cultural and societal concepts have on the context of our experiences.^10^

Data will be analysed using Braun and Clarke’s phase reflexive thematic analysis approach.^9^

1. Familiarisation phase: this involves reading and rereading the data to become immersed in and intimately familiar with its content.
2. Coding phase: this involves generating succinct labels (codes) that identify important features of the data that might be relevant to answering the research question.
3. Generating initial themes: this phase involves examining the codes and collated data to identify significant broader patterns of meaning (potential themes). Then data are collated relevant to each candidate theme.
4. Reviewing themes: candidate themes are checked against the dataset to determine that they tell a convincing story of the data and answer the research question.
5. Defining and naming themes: for each theme, this phase involves developing a detailed analysis, working out the scope and focus and determining the concept.
6. Writing up: this final phase involves weaving together the analytic narrative and data extracts and contextualising the analysis in relation to the existing literature.

Triangulation will be sought from the data within the RCT for concordance between data sets to deepen insights and understandings. Data will be managed with computer-assisted qualitative data analysis software (NVivo V.14 plus).

#### Secondary analysis

A secondary analysis using a large language model will enhance the qualitative analysis. A large language model is a type of artificial intelligence trained using vast amounts of text. These models can generate human-like responses and help simplify complex information. Large language models do not directly access personal information; they learn patterns from combined data. The use of large language models for qualitative data is rapidly expanding in marketing, but few healthcare academics have yet to use this tool. The benefits of using a large language model for qualitative analysis include reduced time and potentially reduced bias. Transcripts will be uploaded into a large language model. Prompts will be developed by a member of the research team (SKM) to provide context to the large language model. The prompts will follow a model of constraining the model using a thematic analysis with a phenomenological underpinning. Context regarding the researcher will also be used as a prompt. The prompts will follow a ‘you are’, ‘I am’ and ‘I want you to’ approach. Prompts will be peer-reviewed by a researcher with experience using large language models for qualitative analysis. Transcripts will be anonymised prior to being entered into the large language model.

The value, efficacy and practicality of using large language models to support qualitative analysis will be considered. These factors will include:

- Percentage of patients providing consent for their data to be used in the secondary analysis.
- A comparison of theme outputs between traditional models and using large language models.
- Data validation.
- Time efficiency: time taken to analyse themes with and without large language models.
- Consideration of bias and whether this is reduced or increased by the addition of the large language model being employed in the analysis.

### Patient and Public Involvement and Engagement

A PPIE will meet quarterly to provide input into the trial’s conduct. Service users were actively involved in the research design; they provided feedback on the proposed methodologies and dissemination plan and coproduced the lay summary. PPIE members provided feedback and ideas on reducing the burden of participating in research and codesigned the recruitment strategy. PPIE members reviewed the study literature, including the participant information sheet and consent forms. The PPPI group reviewed and advised on the topic guide and participated in the pilot.

#### Consent

Participants who are unable to provide informed consent are excluded from the study. The research team will obtain consent before the 1:1 semistructured interview and after the participant’s information sheet review. Potential participants will be given at least 24 hours to consider if they wish to participate in the study. Screening data will be collected regarding the number of eligible participants who agree or decline to participate and the acceptance rates of participating in the secondary analysis.

#### Sponsor

UCL Joint Research Office will act as the sponsor.

#### Audits and inspection

Trial-related monitoring, audits, research ethics committee review and regulatory inspections will be conducted in line with the UK policy framework for health and social care research.^11^

##### Trial management

A trial management group will meet every 6 weeks and monitor all aspects of the conduct and progress of the trial, ensure the protocol is adhered to and take appropriate action to safeguard participants in the quality of the trial. The trial steering committee (TSC) will provide overall supervision of the trial and ensure it is conducted following the principles of Good Clinical Practice and the relevant regulations. The TSC will meet every 6 months during the time of the trial. A Data Management and Ethics Committee will meet annually to provide oversight on the need for any interim analysis and ensure participants’ safety by reviewing recruitment, adverse events and trial completion rates.

##### Publication policy

The results of this clinical investigation will be submitted for publication as an abstract at appropriate conferences and as a journal article to an appropriate journal. Authorship will be determined in accordance with the International Committee of Medical Journal Editors authorship guidelines and UCL publication policy.

##### Timelines

The trial start date was January 2023, with an end date of May 2025 and anticipated publication date of December 2025.

### Rigour

#### Reflexivity

Reflexivity represents how researchers critically evaluate how their beliefs and experiences shape and affect their research. It involves a heightened level of self-awareness.^10 12^ Reflexivity encourages consistent, coherent and transparent engagement with the philosophical underpinning of qualitative research^1013^,^15^ and is a core component of Braun and Clarke’s reflexive thematic analysis method. Reflexivity also aligns with the Heideggerian phenomenological approach, particularly the concept of Being (Dasein), which acknowledges that researchers are not detached observers but are part of the world they are studying.^5^ Reflexivity involves a deep engagement with one’s existence and experience and is thus existential,^16^ which aligns with Heidegger’s emphasis on the lived experience. Heidegger suggests that disruption in our everyday experience is required to make us more aware of our ways of Being;^5^ in concordance with this theoretical concept, reflexivity can disrupt usual ways of thinking and open new possibilities for understanding. Reflexivity is, therefore, a core component of the methods utilised in this qualitative study.

The principal investigator (SKM), a female consultant physiotherapist and PhD student, will conduct the interviews. Participants will have been under the care of the sleep and ventilation service, with direct care provided by the principal investigator, for at least 6 months at the time of the interviews. Thus, there will be an established patient-clinician relationship. Participants will be aware of the research aims and the broader body of work within which this qualitative study is nested.

Reflective logs will be kept to aid the analysis, allow reflexivity and assess researcher preconceptions. Reflective logs will include critical self-reflection, which questions one’s assumptions, biases, and values while considering how these might influence the research. Reflexivity will also be supported with predictive participant responses, whereby the researcher logs anticipated responses before an interview; this method helps to identify researcher preconceptions. Peer debriefing will also be undertaken throughout the research process, including developing methods, piloting, data collection and analysis. Analytical memos will be kept during the analysis phase, enabling documentation of thought processes, decisions and reflections.

#### Trustworthiness and rigour

Several frameworks have been proposed to provide criteria for assessing the quality of qualitative research. Tracy’s eight ‘big tent’ criteria for excellent qualitative research are comprehensive and encompassing.^17^ These criteria include: ‘*worthy topic’*, ‘*rich rigour’, ‘sincerity’, ‘credibility’, ‘resonance’, ‘significant contribution’, ‘ethical’* and *‘meaningful coherence’*. This research has strived to demonstrate high-quality qualitative research in several respects. The topic of the research is meaningful, interesting and justified. Rigour has been shown by clearly articulating the theoretical constructs used throughout the research process. Reflexivity is a core component of the analysis method. Triangulation with quantitative data from the RCT will be deployed where appropriate. Transcripts will be returned to the interviewee to review the transcripts to allow for respondent validation, thus increasing credibility. Procedural ethics have been followed throughout the research process. In addition, the Consolidated Criteria for Reporting Qualitative Research has also been used to ensure the quality of the trial design.^18^

## Discussion

There is a paucity of research considering the lived experience of PAP therapy users, and the limited published data is designed such that results are focused on the negative experiences of PAP therapy. There is a need for a broader approach that encompasses the lived experience of PAP therapy users. The outcomes of this study will provide valuable insights into the lived experience of PAP therapy users and could prove a helpful tool for identifying priority areas for further research.

There are potential limitations to this research. A purposive sample has been chosen to ensure diverse representation, but it may introduce selection bias. Willing participants may have a different lived experience from those not willing to participate; we cannot capture the lived experience of non-participants. The data collection is via semi-structured interviews; the topic guide and underpinning philosophical stance have strived to ensure encompassing and rich descriptions of the lived experience of PAP therapy. However, the data could be subject to recall and social desirability biases. The data collection methods of both face to face and online could add variability; there is a risk that the mode of data collection could affect the depth and nature of the responses. The RCT within which this qualitative study is nested is only 6 months in duration, and all participants are naïve to PAP therapy prior to this point. Thus, this study will not capture the long-term lived experience of PAP therapy.

## Ethics and dissemination

The Hampshire B Research Ethics Committee (REC reference: 22/SC/0405) reviewed the protocol and granted a favourable ethical opinion. The interviews in the qualitative study may cause distress. The interviewer will be trained in identifying the signs of potential distress and will offer the participant the opportunity to pause and/or cease the interview. Mechanisms of support will be implemented for participants, including contact details for the study team and provision of support group details. Ethical considerations of using large language models to support qualitative analysis include the safety and security of participants’ potentially sensitive data, changing terms and conditions of large language models and whether inputted data are used to train the large language model subsequently. Participants will give explicit and optional consent for their data to be included in the secondary analysis, and transcripts will be anonymised prior to entry into the large language model.
