# Supplementary material for: Lived experiences of patients using positive airway pressure (PAP) therapy: a nested phenomenological study within the 3DPiPPIn randomised controlled trial
Source: BMJ Open. 2025 May 6;15(5):e093622. doi: 10.1136/bmjopen-2024-093622 (PMC12056652; doi:10.1136/bmjopen-2024-093622)
Supplement: Supplementary file 1 [file bmjopen-2024-093622.reviewer_comments.pdf]

PEER REVIEW HISTORY

BMJ Open publishes all reviews undertaken for accepted manuscripts. Reviewers are asked to complete a checklist review form and are provided with free text boxes to elaborate on their assessment. These free text comments are reproduced below.

ARTICLE DETAILS

Title (Provisional)

The Lived Experiences of Patients Using Positive Airway Pressure (PAP) Therapy: A Nested Phenomenological Study within the 3DPiPPIn Randomised Controlled Trial

Authors

Mansell, Stephanie K.; GOWING, FRANCESCA; Hilton, Stephen; Main, Eleanor; Mandal , Swapna; Schievano, Silvia; Kilbride, Cherry

---

VERSION 1 - REVIEW

**Reviewer**	**1**
**Name**	**Parthasarathy, Sairam**
**Affiliation**	**The University of Arizona**
**Date**	**12-Apr-2025**
**COI**	**None**

Mansell et al aim to understand the lived experiences of Positive Airway Pressure (PAP) therapy users with the rationale that most published data in this area explores negative experiences. They propose conducting 1:1 semi-structured interviews and rigorous analysis of the qualitative data derived from an ongoing randomized controlled trial investigating the medium-term clinical impact of customized interfaces for patients requiring PAP therapy. They reason that their approach will contribute to a more holistic understanding of the lived experience of PAP therapy users and thereby inform clinical practice and research. The approach for the proposed qualitative study is rigorous but unfortunately the rationale is misleading. I have only one major comment.

Major comment:

There are other qualitative studies in this area that appear to not be cited and therefore do not support the investigators' claim that there is lack of evidence in this area (Brown et al, Sleep. 2021 Oct 11;44(10), Simon et al, J Clin Sleep Med. 2024 Mar 1;20(3):407-416, Waldman et al Health Qual Life Outcomes. 2020 May 7;18(1):128.). Some of these qualitative studies do indeed explore the positive experiences of patients using PAP therapy. The investigators need to contextualize their statements with a more thorough review of the literature and how their approach distinguishes itself from such prior studies.

VERSION 1 - AUTHOR RESPONSE

Reviewer: 1

Dr. Sairam Parthasarathy, The University of Arizona

Comments to the Author:

Mansell et al aim to understand the lived experiences of Positive Airway Pressure (PAP) therapy users with the rationale that most published data in this area explores negative experiences. They propose conducting 1:1 semi-structured interviews and rigorous analysis of the qualitative data derived from an ongoing randomized controlled trial investigating the medium-term clinical impact of customized interfaces for patients requiring PAP therapy. They reason that their approach will contribute to a more holistic understanding of the lived experience of PAP therapy users and thereby inform clinical practice and research. The approach for the proposed qualitative study is rigorous but unfortunately the rationale is misleading. I have only one major comment.

Thank you for your time and energy in reviewing our protocol manuscript. We appreciate your contributions at a time when it is difficult to secure peer reviewers.

Major comment:

There are other qualitative studies in this area that appear to not be cited and therefore do not support the investigators' claim that there is lack of evidence in this area (Brown et al, Sleep. 2021 Oct 11;44(10), Simon et al, J Clin Sleep Med. 2024 Mar 1;20(3):407-416, Waldman et al Health Qual Life Outcomes. 2020 May 7;18(1):128.). Some of these qualitative studies do indeed explore the positive experiences of patients using PAP therapy. The investigators need to contextualize their statements with a more thorough review of the literature and how their approach distinguishes itself from such prior studies.

Thank you for your feedback and the suggested studies for inclusion in the introduction. We have restructured the introduction accordingly. We have included the Brown et al(1) reference. Waldman et al(2) focuses specifically on the burden of excessive daytime sleepiness, and we consider this one aspect of OSA to be too narrow to include in our introduction; it will be included in the results when they are published. Simon et al(3) is a recent publication which was not available at the time of

writing the protocol paper, we have subsequently included this recommendation in the introduction. We have amended the focus of the justification for the study to acknowledge that the research to date has focused on PAP therapy adherence, and by not accounting for the whole lived experience, our understanding becomes constrained. This limited understanding could mean that current clinical pathways are misaligned with the lived experience. Qualitative research, particularly phenomenology, facilitates the amplification of the patient voice. There is a need for further qualitative research that aims to fully encompass the entirety of the lived experience of PAP therapy users.

References

1. Brown A, Jones S, Perez-Algorta G. Experiences of using positive airway pressure for treatment of obstructive sleep apnea: a systematic review and thematic synthesis. Sleep. 2021;44(10).

2. Waldman LT, Parthasarathy S, Villa KF, Bron M, Bujanover S, Brod M. Understanding the burden of illness of excessive daytime sleepiness associated with obstructive sleep apnea: a qualitative study. Health Qual Life Outcomes. 2020;18(1):128.

3. Simon SL, Stephenson JJ, Haynes K, Kennedy ER, Frydman G, Amdur A, et al. The lived experience of positive airway pressure therapy in patients with obstructive sleep apnea across the lifespan: a qualitative study. Journal of clinical sleep medicine : JCSM : official publication of the American Academy of Sleep Medicine. 2024;20(3):407-16.
